# Novel plasma telomerase detection method to improve cancer diagnostic assessment

**DOI:** 10.1371/journal.pone.0174266

**Published:** 2017-05-03

**Authors:** George Hilal, Ruth Reitzel, Zainab Al Hamal, Anne-Marie Chaftari, Iba Al Wohoush, Ying Jiang, Ray Hachem, Issam I. Raad

**Affiliations:** 1 Cancer and Metabolism Laboratory, Faculty of Medicine, Campus of Medical Sciences, Saint-Joseph University, Riad el Solh, Beirut, Lebanon; 2 Department of Infectious Diseases, Infection Control and Employee Health, the University of Texas MD Anderson Cancer Center, Houston, Texas, United States of America; University of South Alabama Mitchell Cancer Institute, UNITED STATES

## Abstract

**Background:**

The activity levels of telomerase and its mRNA have been found to be more diagnostically sensitive than cytological results in many cancerous tissues and correlate well with the clinical disease stage. Currently, there are several methods of detecting telomerase in tissues and in blood. The most commonly used method is a conventional quantitative real-time polymerase chain reaction (PCR) which is time and labor exhausting.

**Methods:**

We have developed a simple and innovative blood test method that allows us to diagnose cancer and relapsed cancer in a cost- and time -effective manner. We had evaluated our novel method in two populations: 1) in vivo in three mice with pancreatic ductal adenocarcinoma (PDAC) versus one control mouse and 2) clinically in 30 cancer patients versus 10 individuals without cancer. We compared our novel method with the old conventional method. At least one sample was obtained from each patient included in the study.

**Results:**

The novel method substantially increased the sensitivity (from 37% to 77%, *p*<0.001) and negative predictive value (from 32% to 56%, *p* = 0.005) of the telomerase test for all cancer patients (those who were substantially treated and those who were not). There was no significant difference in telomerase activity between cancer patients and healthy volunteers using the conventional method (*p* = 0.13), whereas there was a significant difference using the novel method (*p* = 0.001).

**Conclusion:**

Conventional method shows no significant difference in telomerase activity between cancer patients and healthy volunteers (p = 0.13), whereas there was a significant difference using the novel method (p = 0.001).

## Introduction

The activity levels of telomerase and its mRNA have been found to be more diagnostically sensitive than cytological results in many cancerous tissues and correlate well with the clinical disease stage [1,2]. Circulating tumor-derived telomerase RNA was first reported to be detectable in the plasma and serum of patients with nasopharyngeal carcinoma [3] and melanoma[4]. Since then, a number of RNA targets have been detected in patients with breast cancer [5,6], colorectal cancer [7], follicular lymphoma [8], hepatocellular lymphoma, gynecologic malignancies [9], and hepatocellular carcinoma. Telomerase activity shows considerable potential through last decades to be used as cancer biomarker [10,11]. Great efforts have been made over the past decade to detect the two major subunits of telomerase: (1) the human telomerase reverse transcriptase (hTERT) and [2] the human telomerase RNA (hTR). Currently, there are several methods of detecting telomerase in tissues and in blood. The most commonly used method is the conventional quantitative real-time polymerase chain reaction (PCR). RNA telomerase methods, on the other hand, are more complex reverse transcriptase methods that are used to qualitatively detect hTERT and hTR. Both methods are sensitive, but time-consuming and labor- exhausting. Furthermore, sample preparation requires careful handling and a fully trained technician. However, none from the above mentioned methods detect the telomerase activity.

We have developed a simple and innovative blood test method and that allows us to diagnose cancer and relapsed cancer in a cost- and time -effective manner. This quantitative molecular method, which represents a major modification of the conventional method and a major breakthrough in the field of oncology, improves and enhances the detectability of telomerase activity in blood. This novel method is simple, easy and can be completed in a few hours.

The purpose of this retrospective study was to determine the effectiveness of our new method at early diagnosing cancer, relapse, estimating prognosis, and predicting response to therapy, in comparison with conventional methods. Large number of patients with various types and stages of cancer as well as a large number of control patients with no cancer would be necessary.

## Methods

We compared telomerase activity in plasma samples using the Allied Biotech Quantitative Telomerase Detection (QTD) kit and our novel, detection enhancing, modification to the QTD method in two different populations; an in vivo study of residual plasma from mice with pancreatic ductal adenocarcinoma (PDAC) compared to residual plasma from normal control mice, and a clinical study assessing telomerase activity in residual plasma samples from 30 cancer patients compared to 10 individuals without cancer. The study was reviewed and approved by our institutional review board (U.T. MD Anderson Cancer Center Institutional Review Board) (IRB-2) before the study began (protocol LAB09-0310). Blood samples were collected as part of routine medical care. However, this was a retrospective study, and we had no direct contact with patients, and did not collect the samples. A Waiver of Informed consent was approved by the IRB, and we were not required to obtain either verbal or written patient consent.

### Validation of telomerase

#### In vivo study of plasma from mice with PDAC

Residual plasma from banked plasma samples from healthy control mice (no tumor) and mice with pancreatic ductal adenocarcinoma (PDAC) were provided by the study collaborators and was collected for research purposes only. The source of the banked residual mouse plasma samples were obtained from a separate approved investigor project. To minimize telomerase degradation, samples were stored frozen at -80°C until use.

#### Clinical study of plasma from cancer patients and healthy controls

The 30 cancer patients were identified based on the type of cancer to include 20 solid tumors and 10 hematological tumors. Patients were further stratified by status of cancer treatment (untreated, partially treated, substantially treated, and successfully treated) for comparisons of plasma telomerase activity. Residual plasma samples from blood drawn for routine clinical labs were obtained from the MD Anderson Clinical Chemistry after all routine testing had been completed. As control population, whole blood was collected into heparin containing tubes from 10 healthy volunteer employees with no history of cancer from MD Anderson Cancer Center. Whole blood was then processed by centrifugation to separate plasma cells. All plasma was filtrated using a 0.45-mm-syringe filter to eliminate any activated lymphocytes that could cause a false-positive increase in telomerase activity [9]. Filtered plasma was then stored at -80C until use. At least one sample was obtained from each patient included in the study. All samples were tested in duplicates.

#### Activity of telomerase

Plasma samples were tested for telomerase activity using the Allied Biotech QTD Kit (Allied Biotech Inc, Vallejo, CA). Three different sample processing methods (Convention Method, Novel Dialysis Method, and Novel Method 1:1LB–mouse only) were compared to assess potential enhancement of detection of telomerase activity.

Telomerase activity from plasma samples tested by the “Conventional Method” was quantified based on the kit instructions. Briefly, plasma samples were tested by real-time PCR (ABI Prism 7000 Sequence Detection System, ThermoFisher Scientific, Waltham, MA) with SYBR Green dye (QTD master mix) to determine a Ct value for telomerase activity. Ct values were then compared to the standard curve generated by standards provided by the QTD kit to calculate telomerase activity in the unknowns.

The “Novel Dialysis Method” employs extra processing steps to remove any potential substrates that inhibit telomerase detection prior to quantitation with the QTD kit. Aliquots (50uL) of plasma were treated with 7.5 μL of 50% ammonium sulfate and incubated on ice for 25 minutes. Samples were then spun at 14,000 rpm at 4°C for 20 minutes to pellet any precipitated protein. The resulting supernatant was transferred to a 2,000 MW dialysis cartridge and dialyzed for 2 hours at 4°C in 2 L of dialysis buffer (130 mM NaCl, 15 mM MgCl_2_, and 10 mL Tris HCl) to remove any remaining salts. The resulting dialyzed plasma samples were then tested by qPCR for telomerase activity according to QTD Kit instructions.

To further examine the enhanced telomerase detection, dialysis a subset of subsets of samples (mouse only) was treated with 50uL of Lysis Buffer (from QTD kit) incubated on ice for 30 minutes. Samples were then spun at 14,000 rpm at 4°C for 20 minutes. The resulting “Novel Method 1:1 LB” samples were then tested by qPCR for telomerase activity according to QTD Kit instructions.

## Statistical methods

Wilcoxon rank-sum test was used to compare telomerase activity levels between cancer patients and their healthy controls. The optimum cut-off value was determined for each method on the basis of the Youden index, a function of sensitivity and specificity, which is commonly used to measure overall performance of a diagnostic test [12]. With the cut-off value chosen, sensitivity, specificity, positive and negative predictive values were estimated. McNemar’s test was used to compare sensitivity and specificity. A score statistic derived from a marginal regression model was used to compare predictive values [13]. All tests were two-sided at a significance level of 0.05. The statistical analyses were performed using SAS version 9.3 (SAS Institute Inc., Cary, NC).

## Results

### Validation of telomerase measurement

**Fig 1:**
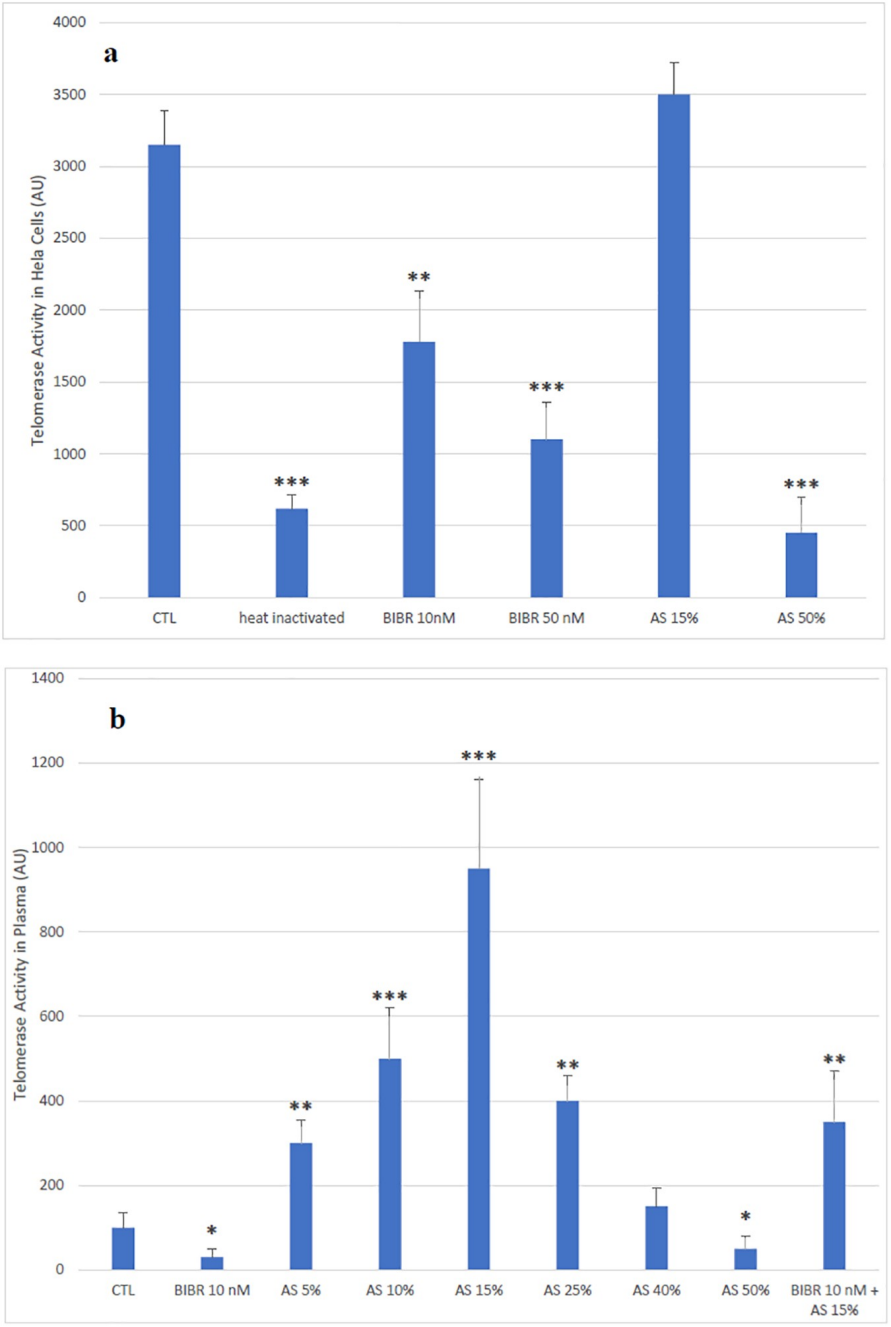
Validation of the new method using HeLa cells and plasma. (A) HeLa cells were purchased from ATCC and cultured in DMEM and 10% FBS, 1% penicillin /Streptomycin and incubated at 37oC in 5% CO2 incubator. Cells were lysed according to the manufacture instructions. Telomerase inhibitor BIBR-1532 was directly added to the sample. Heat inactivation of the telomerase was performed by incubating the sample in boiling water for 10 minutes. Samples not treated with ammonium sulfate received an equal amount of water. (B) Effect of BIBR-1532 and ammonium sulfate at different concentrations on plasma telomerase activity. The unpaired Student test was performed comparing to control (* P<0.05; ** P<0.01; ***P<0.001)

In order to validate telomerase activity measurement and discard any artifact in our results, we applied the pre-measurement treatment to the HeLa cells extract, heat inactivated the enzyme, and directly inhibited the proteic subunit hTERT with BIBR-1532. Fig 1A shows that the activity of the BIBR-1532 and cell extract boiling decreased the activity measured by the technique, however, the ammonium sulfate at 15% did not show any effect while the 50% concentration significantly inhibited telomerase activity. The Fig 1B shows a dose-dependent effect of plasma pretreatment with ammonium sulfate. The results show that at low concentrations of ammonium sulfate, telomerase remains active while at high concentration the enzyme precipitates with proteins and becomes inactive. In addition, the activity was inhibited by BIBR-1532; the heat-inactivation was not possible with plasma since proteins concentration is very high and precipitate with heat.

### Telomerase activity in mice with PDAC

**Fig 2:**
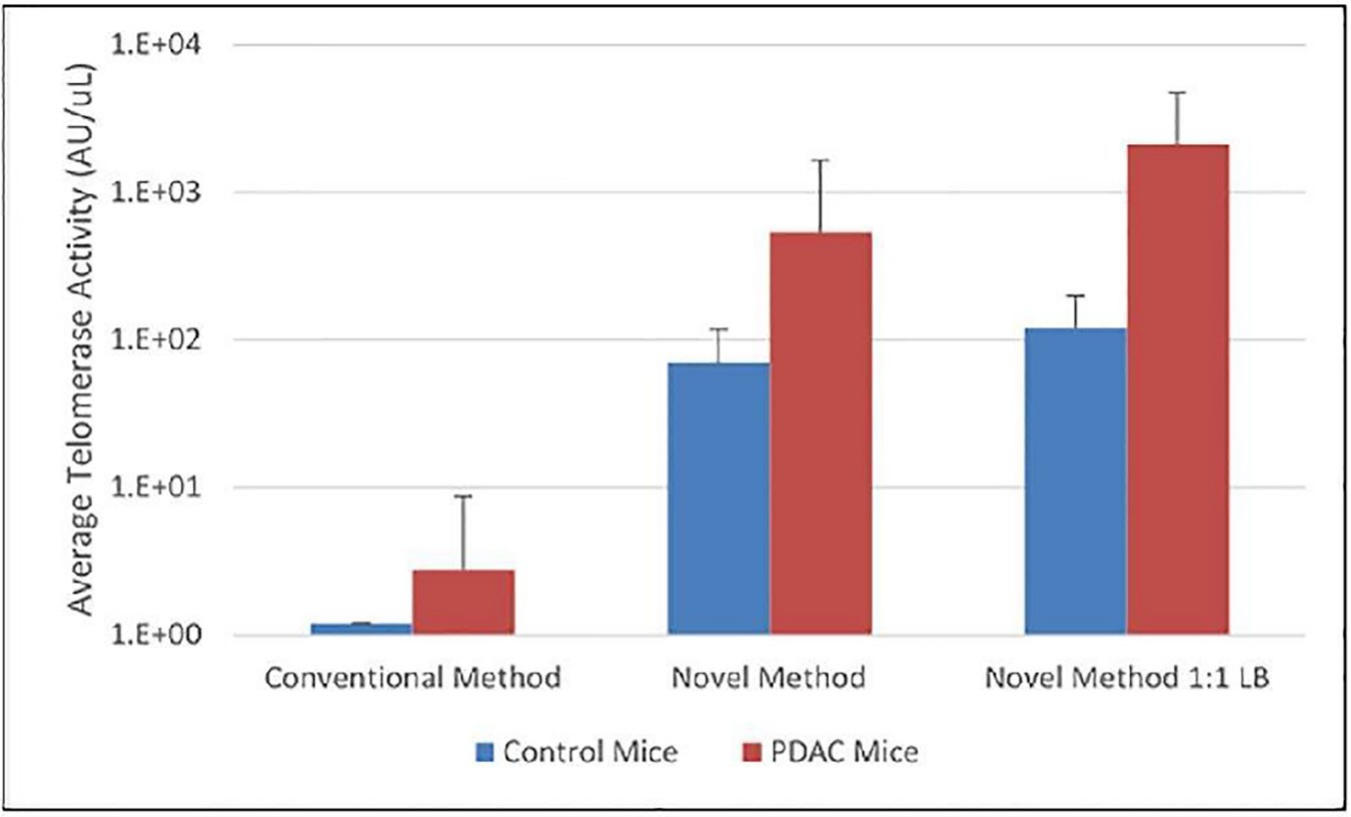
Comparison of the activity of Telomerase with three detection methods. The conventional method, novel method, and novel method 1:1LB were used to detect telomerase activity in control mice and PDAC mice. Novel Method 1:1:LB enhances the detection of telomerase compared to both novel and conventional methods.

Telomerase activity in plasma detected by the Conventional Method, Novel Method, and Novel Method 1:1 LB was compared for 3 control mice (healthy), and 7 mice with PDAC. Using the conventional method, telomerase activity was not detected in any of the control mice and in only 2 of 7 cancer mice. Further investigation showed that the two PDAC mice that showed telomerase detection using the conventional method had an abundance of lysed red blood cells in the sample which may have resulted in a false positive. Use of the Novel Method and Novel Method 1:1 LB increases the average telomerase activity in both control and cancer mice (Fig 2). Despite the increase in background telomerase detection in control mice, the differentiation in telomerase activity between cancer and control mice is also greater. We detected a 1.24 log_10_ difference between cancer and control mice using the Novel Method 1:1 LB compared to a 0.89 log_10_ and 0.44 log_10_ difference in the Novel Method and conventional methods, respectively.

**Fig 3:**
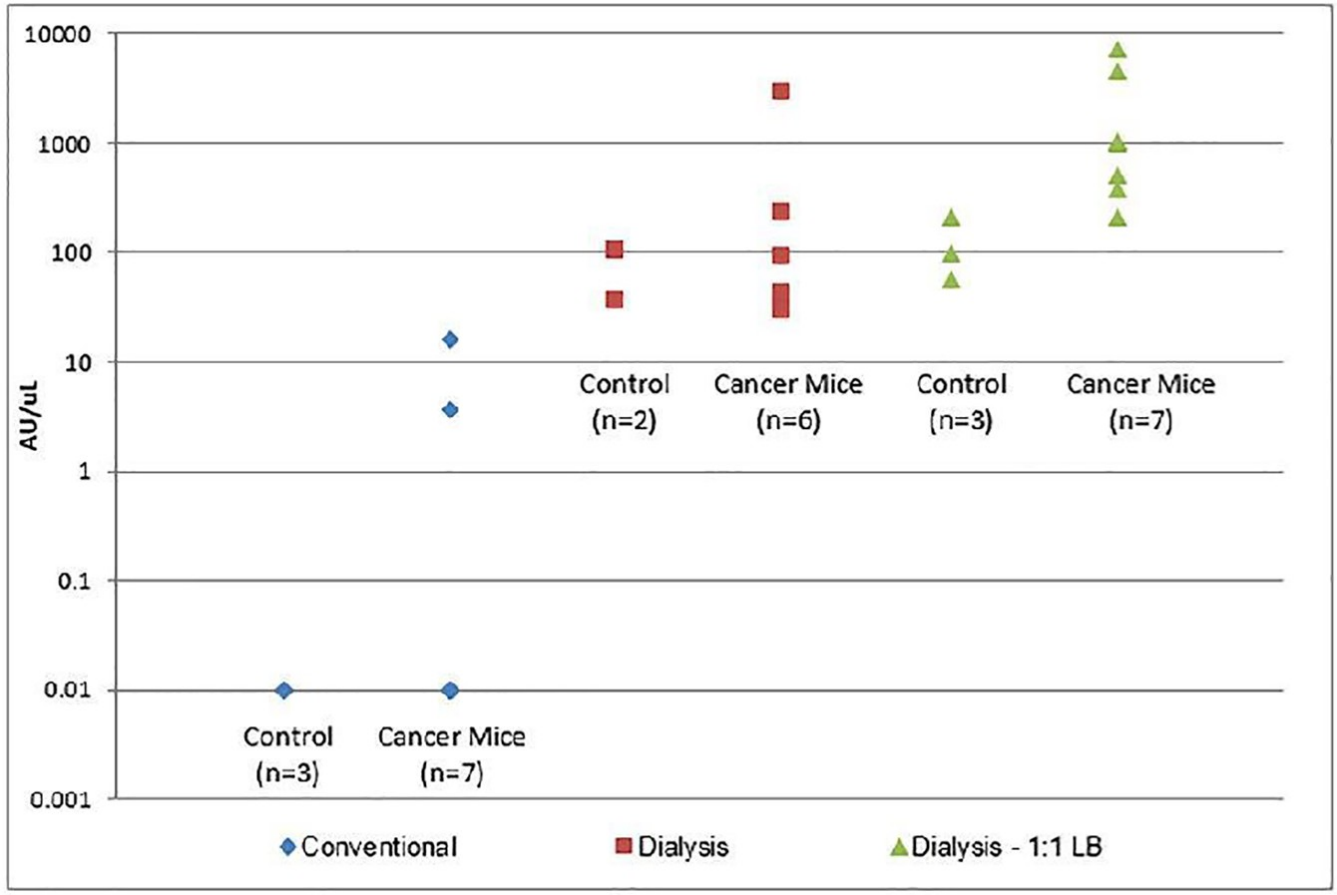
Ranges of Telomerase levels of control and PDAC cancer mice. Undetectable samples using the conventional method (2 control, 5 cancer mice) are denoted as 0.01 AU which is lower than the detectable limit of the commercial assay.

To further compare the benefit of the Novel Method or Novel Method 1:1LB Fig 3 shows ranges of telomerase detection from control and PDAC cancer mice. With the conventional method the majority of samples (2 control, 5 cancer) are undetectable. These are displayed as a simulated 0.1AU/uL which is below the limit of detection of the commercial QTD assay. In the samples treated with the Novel Method 1:1LB there is distinction with very little overlap between the control and cancer mice in comparison with the Novel Method where several of the samples overlap. This indicates that the use of both the Novel Method and Novel Method 1:1 LB enhances the ability to detect and differentiate telomerase activity in mice with cancer vs no-cancer.

### Telomerase activity in cancer patients

**Fig 4:**
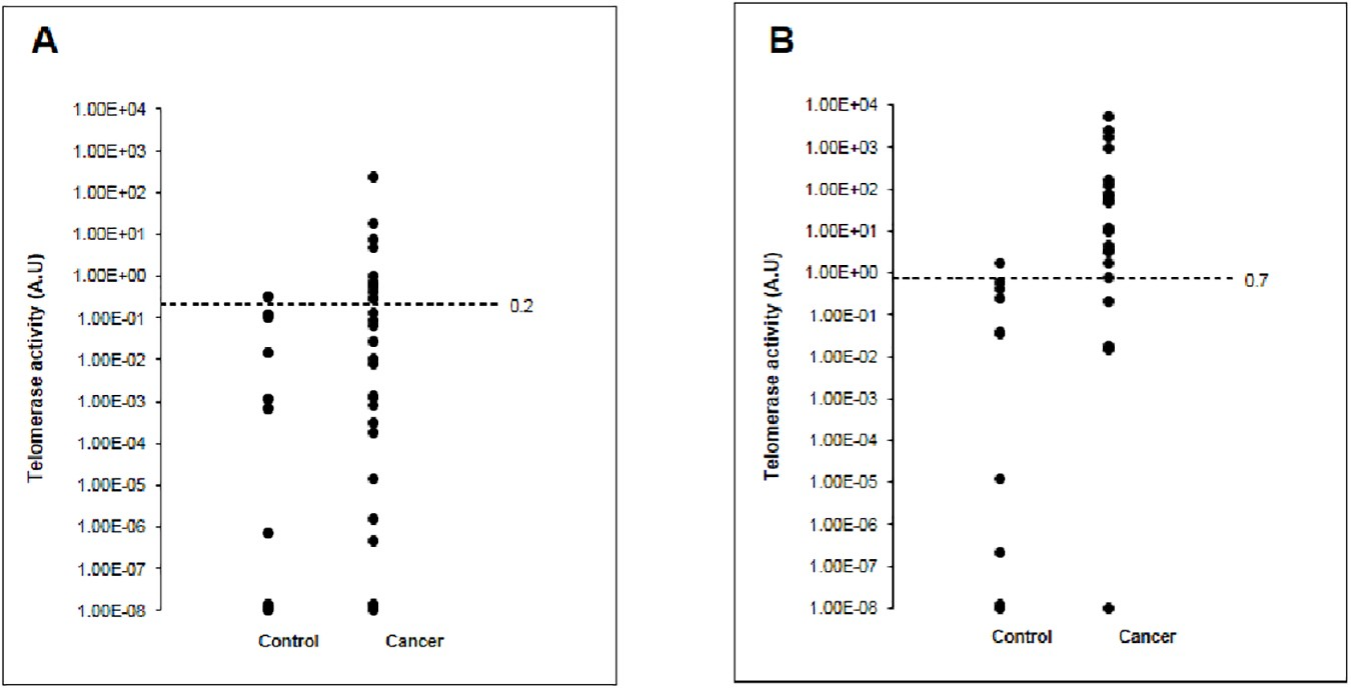
Activity of Telomerase in Cancer Patients and Healthy Controls. Activity of telomerase was detected in plasma of cancer patients and healthy controls using two different methods, Conventional method (A), and Novel Method (B).

There was no significant difference in telomerase activity between cancer patients and healthy volunteers using the conventional method (median level of telomerase activity: 0.028 A.U/ul vs 0.001 A.U/ul, *p* = 0.13) (Fig 4A), whereas there was a significant difference between the two groups using the novel method (median level of telomerase activity: 10.71 A.U/ul vs 0.037 A.U/ul, *p* = 0.001) (Fig 4B).

**Fig 5:**
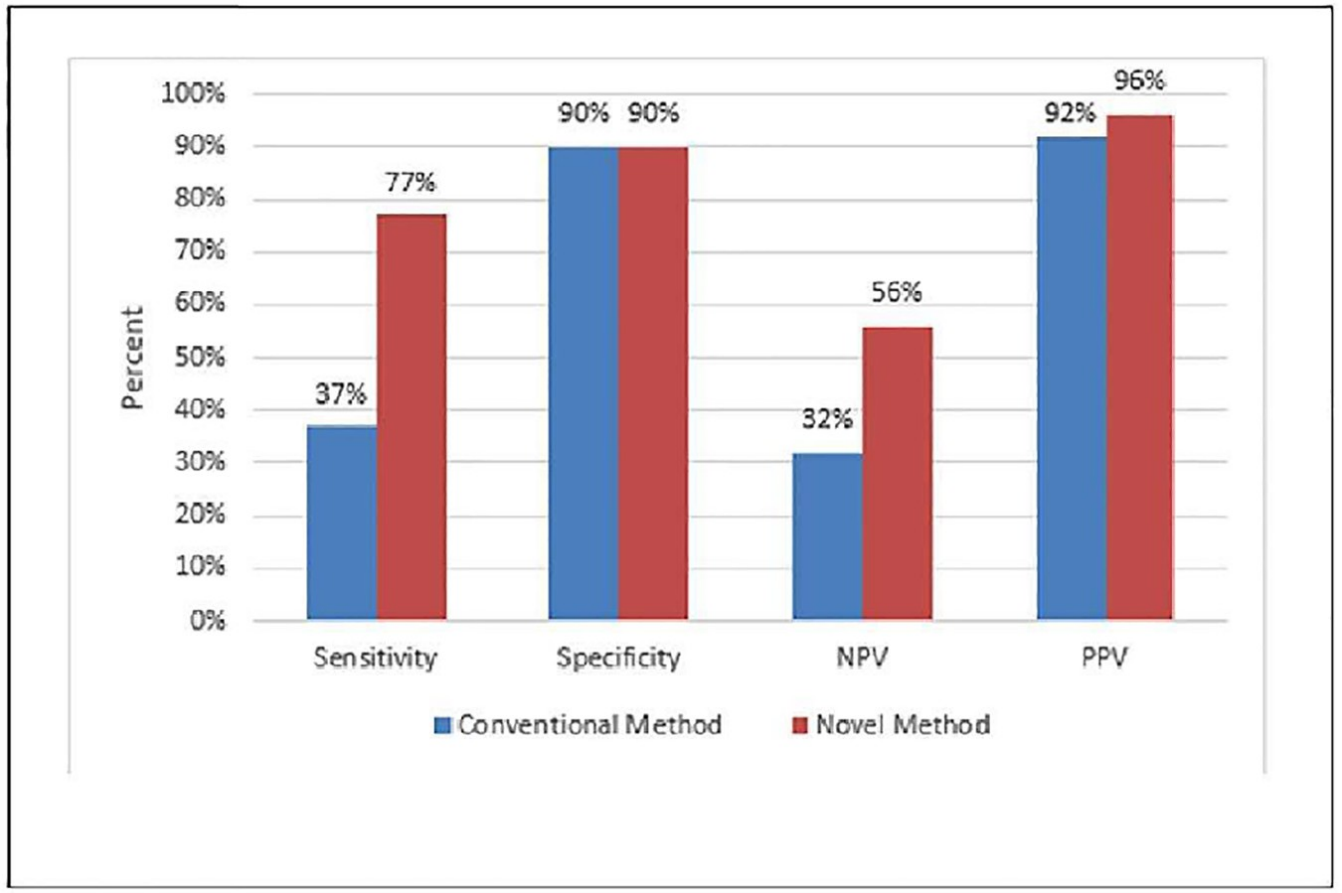
Diagnostic performance of novel vs conventional telomerase detection methods for all cancer patients.

We further compared the diagnostic performance of the two methods. With cut-off value of 0.7 A.U/ul, the novel method had a sensitivity of 77%, specificity of 90%, positive and negative predictive values (PPV and NPV) of 96% and 56% for cancer diagnosis, showing a significant improvement in sensitivity (*p*<0.001) and NPV (*p* = 0.005) when compared to the conventional method which had a sensitivity of 37%, specificity of 90%, PPV of 92% and NPV of 32% with cut-off value of 0.2 A.U/ul. (Fig 5) The performance improvement of the telomerase test was for all cancer patients, including those who were substantially treated and those who were note.

## Discussion

Cancer diagnosis, either at early stages, before and after treatment using a simple blood test, remains a big challenge for the scientific community. A lot of efforts have been deployed in order to find sensitive markers; however, almost all the classical cancer markers presently used for diagnosis are not very sensitive nor specific and are also expressed in benign conditions. The discovery of telomerase gave hope to achieve this goal. The ultimate goal of this study was to assess the telomerase activity measurement in serum and/or plasma in cancer patients and normal individuals. The quantitative real-time PCR technique failed to measure any telomerase activity in plasma above the detection limit of the technique. Trying to overcome this obstacle, the plasma was treated with 15% from ammonium sulfate. At this low concentration, the salt solubilizes proteins, separate binding proteins however doesn’t precipitate it. After treatment, the salt was eliminated by dialysis. This treatment significantly increased telomerase activity measured in plasma compared to non-pre-treated plasma. Despite the diagnostic utility of this promising modified technique, the treatment of plasma with ammonium sulfate lead us to a couple of observations: first, the source of telomerase activity in plasma could most probably be the tumor itself, since the activated lymphocytes and the circulating tumor cells, that express telomerase, are discarded by centrifugation and filtration; second, telomerase in plasma is most likely bounded to a protein that hides its catalytic subunit of the enzyme, which is not the case with HeLa cells since the pre-treatment of the extract with ammonium sulfate at 15% did not increase the telomerase activity. More investigations are needed in order to elucidate the nature of this behavior. Similar finding was observed in the detection of alpha-fetoprotein in the cytosol of human breast cancer. However, Sarcione et al, [12] observed that the alpha-fetoprotein was bounded to the estrogen receptor in breast cancer cell lysate and a salt treatment with KCl was required to detect the protein by radioimmunoassay.

Telomerase activity was first measured in mice serum with pancreatic cancer. The results showed clearly that the new method, but not the conventional one, detected telomerase activity in cancerous serum mice whereas this activity was not detectable in serum from normal mice. This observation strongly suggests that this new method detects telomerase and the origin of the enzyme is the tumor itself. The work on patients’ serum demonstrated that the sensitivity of the new method is significantly higher than the conventional method (77% vs 37% respectively), but not the specificity which is 90% for both methods. While the sensitivity is comparable to the sensitivity of the classical tumor markers [13], the specificity of this technique doesn’t inform about the origin of the tumor however the information is valid to detect the presence of cancer. The PPV of the new test is excellent (96%) while the NPV is relatively low (56%). This means that the telomerase activity test should be combined to another tumor marker not to miss true positive patients. This low NPV is due either to very small tumors, tumors that use alternative lengthening of telomere (ALT) instead of telomerase to maintain telomere length or to a remission after treatment. Our results also showed that the novel method further discriminated cancer patients on the basis of the extent of and response to therapy, which has major prognostic and therapeutic implications.

Since the discovery of telomerase until the beginning of the new millennium, all the efforts on telomerase activity studies were limited only to tissues, especially to cancerous tissues and body fluids [14,15]. These studies revealed that telomerase is more sensitive than cytology, which is considered the gold standard, and its activity measurements have two utilities: first the early detection marker of cancers that upregulate telomerase and second the prognosis indicator. The telomerase specificity for cancer lead further works to detect the hTERT mRNA, the catalytic unit of the enzyme, in blood and serum. The sensitivity and the specificity are 70% and 90% respectively in non-small cell lung cancer[16], in gall bladder and carcinoma [17], in bladder cancer [18,19], in breast cancer [20,21], in hepatocellular carcinoma [22–24], prostate cancer [25,26], cervical cancer[27], colorectal cancer [28] and gastric cancer [29]. One of the major advantages of our novel technique is the quantification of the active protein and not the mRNA that could be present but not translated into an active protein. In addition, the level of serum telomerase activity may correlate with cancer activity, size [14] and aggressivity [14,30]. Recently, Goldkorn et al [15] published a new method on telomerase activity measurement in circulating tumor cells using a microfilter where cells lyse and telomerase activity is performed using a traditional TRAP assay. Such a method is only valid in metastatic cancers and doesn’t consider telomerase activity in the activated lymphocytes during inflammation that often coexist with cancer [30].

In conclusion, our team developed a new plasma pretreatment technique for telomerase activity measurement. The plasma salt treatment and dialysis increased the sensitivity of telomerase activity detection and made possible the use of the quantitative real time-PCR in cancer diagnosis, to discriminate normal individuals from cancer patients and to assess patient’s response to treatment. While the ultimate aim of our work was to develop a simple and sensitive test, the major limitation of our study is the small number of patients. Another limitation is the lack of clinical inofmration about the patients in the study. The clinical utility of this novel method should be further evaluated in a large prospective study where the plasma telomerase activity will be compared with the appropriate classical markers for each cancer type and followed longitudinally to assess its diagnostic and theragnostic roles.
